# Protein/Lipid ratio of pollen biases the visitation of bumblebees (*Bombus ignitus* Smith) to male-fertile cultivars of the Japanese pear (*Pyrus pyrifolia* Nakai)

**DOI:** 10.1371/journal.pone.0297298

**Published:** 2024-02-26

**Authors:** Shinnosuke Mori, Masahiro Mitsuhata, Tomoyuki Yokoi

**Affiliations:** 1 Faculty of Science and Technology, Keio University, Yokohama, Kanagawa, Japan; 2 Arysta LifeScience Corporation, Chuo-ku, Tokyo, Japan; 3 Graduate School of Science and Technology, University of Tsukuba, Tsukuba, Ibaraki, Japan; National Institute of Agricultural Research - INRA, MOROCCO

## Abstract

Bees have been known to visit the male-fertile cultivars of self-incompatible flowering plants more frequently than the male-sterile cultivars, but the origin of this preference is poorly understood. Here, we demonstrate that this preference is driven by the higher protein/lipid ratio of male-fertile pollen compared with male-sterile pollen by way of two caged-behavioral assays with six cultivars. In the first assay, flower-naïve bumblebees (*Bombus ignitus* Smith) showed a significantly higher flower-visitation rate to male-fertile cultivars (pollen germination rate > 55%; > 14 visits/10 min) of the Japanese pear (*Pyrus pyrifolia* Nakai) than male-sterile cultivars (pollen germination rate ≤ 20%; > 6 visits/10 min). In the second, bees still preferred the anthers of male-fertile cultivars (5–9 visits/10 min) more than those of male-sterile ones (less than 1 visit in 10 min) even in the absence of all other organs (i.e., petals, pistil, nectar), indicating that pollen is responsible for the preference. We then analyzed the macronutrient content of the pollen and its visual cues, and found that the bee preference was highly correlated with the protein/lipid ratio (0.3–1.6) but not color variables such as (a)chromatic contrast, intensity, and spectral purity. We conclude that the protein/lipid ratio influences the foraging behavior of the bumblebees likely by serving as (1) a chemotactile cue while antennating, (2) a gustatory cue after intake, and (3) an olfactory cue. In addition, the low bee visitation rate to poorly viable pollen could be due to its low protein/lipid ratio.

## Introduction

Self-incompatibility, the inability to produce zygotes after self-pollination in a fertile hermaphrodite plant [1], is a characteristic of about half of all flowering plant species, and an effective system to prevent inbreeding and promote genetically diverse populations. However, self-incompatibility impedes stable fruit production in many economically important crops such as Rosaceae fruit trees (e.g., apple, pear, apricot), which are therefore highly dependent on pollinating agents to achieve cross-pollination with a different cultivar (pollinizer) necessary to ensure fruit production. Indeed, Rosaceae fruit trees are usually pollinated by either honeybees (*A*. *mellifera*) or bumblebees (*B*. *terrestris* L., *B*. *ignitus* Smith) intentionally introduced into orchards [2,3], and a field investigation demonstrated that the exclusion of pollination by reared honeybees drastically reduced the yield of all tested cultivars of pear [4]. Due to the large dependence on bees, an understanding of bee behavior in orchards of self-incompatible crops is essential.

Previous studies have noted a bias in the visitation of bees for male-fertile cultivars over -sterile ones of several self-incompatible crops, including Rosaceae fruit trees, such as apricot [5], peach [6], and pear [7]. For example, in a study of the Japanese pear (*Pyrus pyrifolia* Nakai), the pollination frequency of male-fertile cultivars (‘Manpungbae’, ‘Whasan’) by bumblebees (*B*. *terrestris*) was 2.8-times higher compared with that of male-sterile ones (‘Niitaka’, ‘Whangkeumbae’) [7]. However, the characteristics of the pollen responsible for this difference are unclear.

A suite of visual and olfactory cues associated with the color and odor bouquets exhibited by pollen attract pollinating bees [8–10], for whom pollen is a primary source of nutrition, providing proteins, lipids, free amino acids, and carbohydrates for larval development, adult maintenance, and sexual maturation [11]. Recent studies have demonstrated that pollen protein/lipid (P/L) ratio is one such chemotactile cue in host-plant choice, and ruled out the individual influence of the concentration of proteins, lipids, and carbohydrates [12,13]. For example, as the P/L increased, the common eastern bumblebees (*B*. *impatiens* Cresson) visited plant species more frequently, peaking at P/L = 5 and 10. This implies that *B*. *impatiens* workers are not just looking for more protein, but rather trying to balance their nutrition [12]. Furthermore, Kraus et al. (2019) reported that *B*. *terrestris* regulates P/L to different ratios, with an average of 3 in microcolonies [14]. Based on these findings, we hypothesized that male-sterile cultivars produce pollen with low P/L, and that this biases bee visitation to flowers of male-fertile cultivars over that of male-sterile cultivars.

Here, we examined the behavioral preference of flower-naïve bumblebees (*B*. *ignitus*) to six leading male-fertile/sterile cultivars. We then explored inter-cultivar differences in pollen-derived nutrition (P/L) and visual cue responsible for the selective behavior of bumblebees towards male-fertile/sterile cultivars.

## Materials and methods

### Reagents

All reagents were of analytical grade and purchased from FUJIFILM Wako Pure Chemical (Osaka, Japan) and Nacalai Tesque (Kyoto, Japan), unless otherwise stated.

### Plant materials

**Fig 1:**
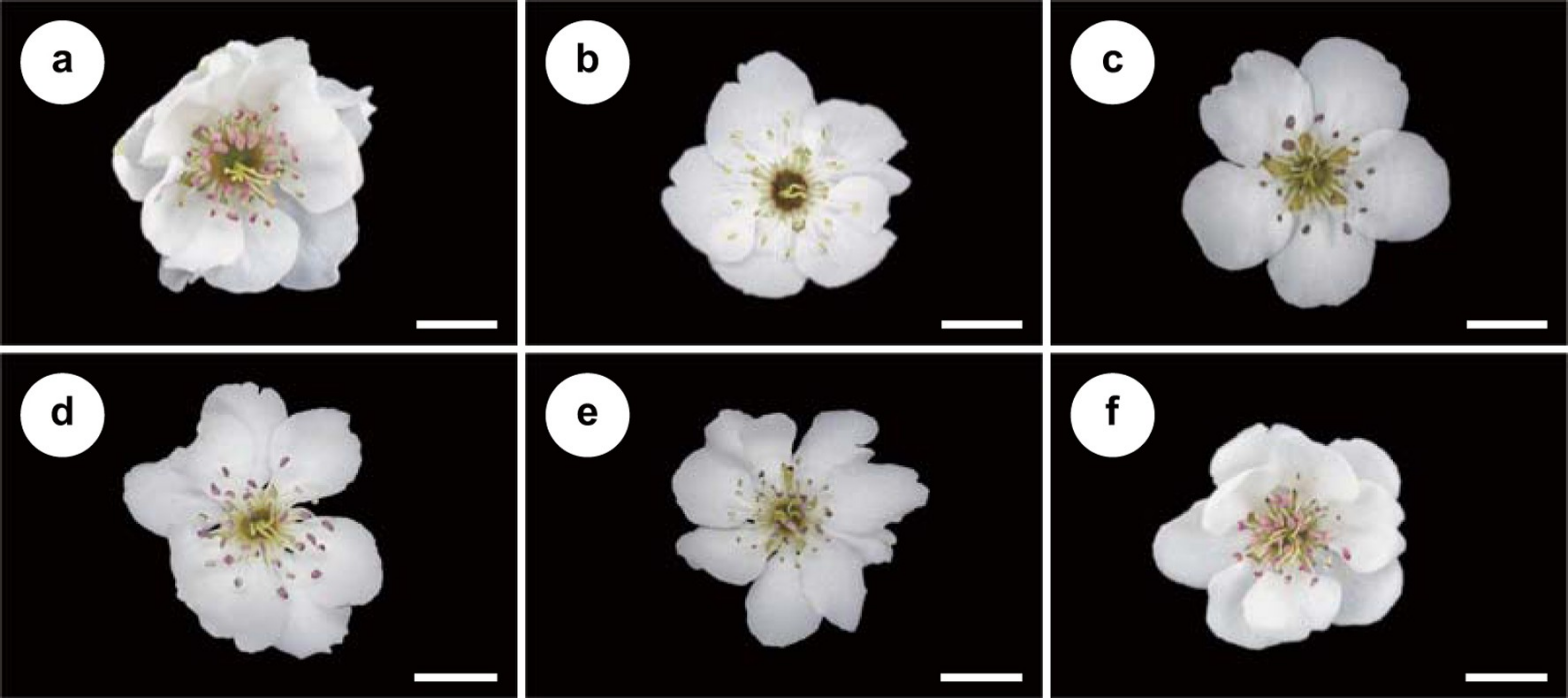
Flowers of the six cultivars of *Pyrus pyrifolia* and their percentage contribution to the total pear-cultivated area in Japan. (**a**) ‘Kosui’ (39.1%), (**b**) ‘Hosui’ (25.9%), (**c**) ‘Niitaka’ (9.3%), (**d**) ‘Akizuki’ (5.5%), (**e**) ‘Shurei’ (0.3%), and (**f**) ‘Inagi’ (0.3%). Scale bars, 10 mm.

Six leading cultivars of *Pyrus pyrifolia* Nakai (Rosaceae, Pyrinae) were used (Fig 1): ‘Kosui’ (syn. ‘Kousui’) (39.1%), ‘Hosui’ (syn. ‘Kousui’) (25.9%), ‘Niitaka’ (9.3%), ‘Akizuki’ (5.5%), ‘Shurei’ (0.3%), and ‘Inagi’ (0.3%), which together account for 80.4% of the total pear-cultivated area in Japan [15]. Of these cultivars, ‘Niitaka’ is characterized by its male-sterility [16]. Fresh flower- and bud-bearing branches of these cultivars were collected from five managed orchards: ‘Kosui’ in Kanagawa (35°53’11.9"N, 139°59’71.9"E); ‘Hosui’ in Kanagawa (35°38’80.7"N, 139°32’32.9"E); ‘Inagi’ in Tokyo (35°63’91.7"N, 139°51’59.0"E); ‘Niitaka’ in Tochigi (36°63’01.0"N, 139°94’53.9"E); and ‘Shurei’ and ‘Akizuki’ in Tochigi (36°55’74.4"N, 139°81’08.3"E) during March and April 2023. After the buds flowered in the laboratory, they were photographed within hours and immediately used for further experiments. Fresh anthers were collected from flower buds at the balloon stage and incubated at 20°C for 10 h to promote dehiscence for behavioral assay, microscopy, and spectral analysis. Pollen grains were collected from the fresh anthers and used for the viability test and nutritional analysis. In the figures and table, the six cultivars are presented in order of ascending size of cultivated area, as described above.

### Field investigation on the fruit set of bee-pollinated pears

A field investigation was conducted in a managed pear orchard (approx. 1,520 m^2^) from March to April 2021 in Saitama, Japan (36°03’69.5"N, 139°59’73.3"E), where the male-fertile and -sterile cultivars were mixed-planted. The orchard comprises a random distribution of 40 ‘Niitaka’ trees (male-sterile), 17 ‘Akizuki’ trees (male-fertile), and ten ‘Kaori’ trees (male-fertile), with four ‘Shinko’ trees (male-fertile) located at the northern, southern, and eastern ends, as pollinizers. Two queenright colonies of the fiery-tailed bumblebee (*Bombus ignitus* Smith; Arysta LifeScience, Tokyo, Japan), a generalist species used in pear orchards in Japan, in hives were introduced into the orchard when it had reached a growth stage of code 63 on the BBCH-scale, corresponding to (about 30% of flowers open) [17]. The number of bumblebee hives per unit area to be introduced into the orchard was determined based on a previous study on *P*. *pyrifolia* [7]. The hives were placed near the base of two of ‘Shinko’ trees. The orchard was covered by nets to exclude any visitation of wild pollinators. Neither artificial pollination nor flower-removal treatments were performed.

The fruit sets of ‘Niitaka’ and ‘Akizuki’ were surveyed at a growth stage of code 72 on the BBCH-scale, corresponding to fruit up to 20 mm in size [17]. Three trees were used for both cultivars. The fruit set was calculated by dividing the number of fruits by the number of total flowers (n = 481 for ‘Niitaka’, n = 552 for ‘Akizuki’). The percentage of inflorescences with one or more fruits among the surveyed inflorescences was also calculated by dividing the number of inflorescence bearing one or more fruits by the number of total inflorescences (n = 104 for ‘Niitaka’, n = 90 for ‘Akizuki’).

### Caged-behavioral assays with bumblebees

Two bumblebee behavioral assays were carried out between 9:00 am and 3:00 pm on the Yagami Campus of Keio University, Japan (35°33’19.6"N, 139°39’16.6"E) over eight days in March 2023 using *B*. *ignitus*. A queenright colony containing approx. 300 flower-naïve *B*. *ignitus* was bred in a hive. Dried pollen and sugar solution were provided *ad libitum* to the bumblebees before and between the assays. The hive was placed in a flight-cage measuring W0.6 × D0.6 × H0.9 m, in which the assay was performed. ‘Kosui’, ‘Hosui’, ‘Niitaka’, ‘Akizuki’, ‘Shurei’, and ‘Inagi’ were used in the assays. The order of cultivars tested was randomized so that the same cultivars were not tested in succession. Both assays were replicated eight times for each cultivar; data are presented as mean ± SD (n = 8).

In the first assay, a branch bearing approx. 100 fresh flowers was placed in the flight-cage, and the number of bee landings on flowers was counted over a period of 10 min. The number was expressed as flower-visitation rate (visits/10 min). The branch was replaced for each test to prevent confounding of the results due to any scent mark and reward consumption. Because ambient solar illuminance can affect bee activity [18], it was recorded during our assays with a light meter LX-1128SD (Lutron Electronic Enterprise, Taipei, Taiwan).

In the second assay, a Petri dish containing about 100 anthers was placed in the flight-cage, and the number of feeding events on the anther was counted for 10 min. The anthers were replaced after each test. The number was expressed as anther-visitation rate (visits/10 min).

### Scanning electron microscopy of pollen

The anthers were fixed by soaking in FAA (formalin-acetic acid-alcohol) fixative (63% EtOH, 5% AcOH, 5% HCHO) at 4°C overnight, then dehydrated in a graded EtOH series and in *t*-BuOH and freeze-dried at −20°C. The dried anthers were mounted on stages using adhesive conductive tape and coated with osmium via hollow cathode plasma CVD (HPC-20; Vacuum Device, Ibaraki, Japan). The anthers were observed by SEM (Miniscope TM3030 Plus; Hitachi High-Technologies, Tokyo, Japan) at an acceleration voltage of 1.5 kV.

The length of the polar axis (P) and the equatorial diameter (E) were measured using ImageJ2 ver. 2.9.0 [19]. P/E ratio was calculated to define their shape class [20]. Ten pollen grains of each cultivar were used for the P/E evaluation. Palynological terminology follows Punt et al. (2007) [20].

### Pollen viability test

Fresh pollen grains were collected from the dehisced anthers, and cultivated on a medium containing 1% agar (w/v), 15% sucrose (w/v), and 0.01% boric acid (w/v) for 10 h in the dark at 20°C. Pollen germination was defined as pollen tube length exceeding the diameter of the pollen grain. A single test was carried out with over 100 pollen grains. The tests were replicated three times. The percentage of pollen germination was then determined by microscopic observation (CKX53; Olympus, Tokyo, Japan). The germination rate is presented as mean ± SD (n = 3).

### Pollen nutritional analysis

Pollen was dried overnight in a desiccator before nutritional analysis. Dried pollen of approx. 1 mg was ground in 300 μL H_2_O using BioMasher Standard (Takara Bio, Shiga, Japan) and vortexed. The aqueous solution was centrifuged at 20,000 ×g for 5 min. The supernatant was subjected to the Qubit Protein Assay, according to Nikkeshi et al. (2021) [21], using a Qubit 3.0 Fluorometer (Thermo Fisher Scientific, MA, USA).

Pollen lipid concentrations were determined using the protocol of Van Handel and Day (1988) [22], with minor modifications. Dried pollen of approx. 1 mg was ground in 0.2 mL of 2% (w/v) Na_2_SO_4_ using BioMasher Standard (Takara Bio). The aqueous solution was transferred to a glass tube, then diluted with 1.6 mL of a 1:1 mixture of MeOH–CHCl_3_, to emulsify the lipids. The tube was then centrifuged for 5 min, and the supernatant transferred to another glass tube to which was added 0.6 mL H_2_O. After vortexing, the tube was then centrifuged for 5 min to separate the layers of MeOH aq. and CHCl_3_. The MeOH aq. layer was removed, and the CHCl_3_ layer was then evaporated *in vacuo* to give a residue which was heated at 110°C in 95% H_2_SO_4_ using an oil bath. Vanillin reagent (1:4 0.6% vanillin (w/v)–85% H_3_PO_4_; 5 mL) was added to the tube, which was then incubated at rt. After 5 min, the absorbance at 525 nm (A_525_) of the mixture was measured in a quartz cuvette with a V-730 spectrofluorometer (Jasco, Tokyo, Japan). The concentration of proteins and lipids are reported as μg/mg DW of pollen. The quantification was conducted with three individual extracts for each cultivar, which were expressed as mean ± SD.

### Diffuse reflectance spectral analysis

Diffuse reflectance spectra of the petals (adaxial side) and dehisced anthers with pollen were measured from 300 nm to 700 nm using a UV-3600 Plus spectrometer (Shimadzu, Kyoto, Japan) equipped with an integrating sphere unit ISR-603 (Shimadzu). The samples were measured in a powder sample cell (Shimadzu) using Spectralon (Labsphere, NH, USA) as a white standard. Data were processed with a software UV-Probe 2.50 (Shimadzu) and are presented as the mean of five biological replicates.

### Visual modeling

The color loci of each petal and anther in the bee’s trichromatic color space were computed using their diffuse reflectance spectra with *pavo* version 2.7.0 [23] in R version 4.1.2 [24]. The daylight irradiance spectrum CIE D65 was used to model daylight irradiance. The reflectance spectrum of green foliage in the *pavo* was assumed as the standard background of the petal and anther. The spectral sensitivity of *A*. *mellifera* was used as a model, due to the conservation of the photopigments underlying trichromatic vision in Hymenoptera [25].

The loci were plotted in the bee color hexagon space (CH model) [26]. The hexagon was divided into six categories based on their stimulation of honeybee photoreceptors: UV, UV-Blue, Blue, Blue-Green, Green, and UV-Green. Color conspicuousness of the flower organs was evaluated with reference to four factors: chromatic contrast, achromatic contrast, intensity, and spectral purity, all of which may influence visual attractiveness. Chromatic contrast, the color contrast without brightness information, was evaluated by the pairwise Euclidean distance between the flower locus and the hexagon center, representing the leaf background, and is given in hexagon units [26]. Achromatic contrast refers to the brightness difference between the flower locus and the leaf background and corresponds to the green photoreceptor excitation, *E* (G), adapted to the background, which was calculated as the absolute value of |0.5 − *E* (G)| as per Spaethe et al. (2001) [27]. Color intensity was calculated as a sum of photoreceptor excitations [27]. Spectral purity refers to the saturation of color and was calculated as the perceptual distance between the flower locus and the background divided by that between the maximal spectral purity locus and the background [28,29]. In *B*. *terrestris* and *A*. *mellifera*, color with higher spectral purity is preferred [30].

### Statistics

**Fig 2:**
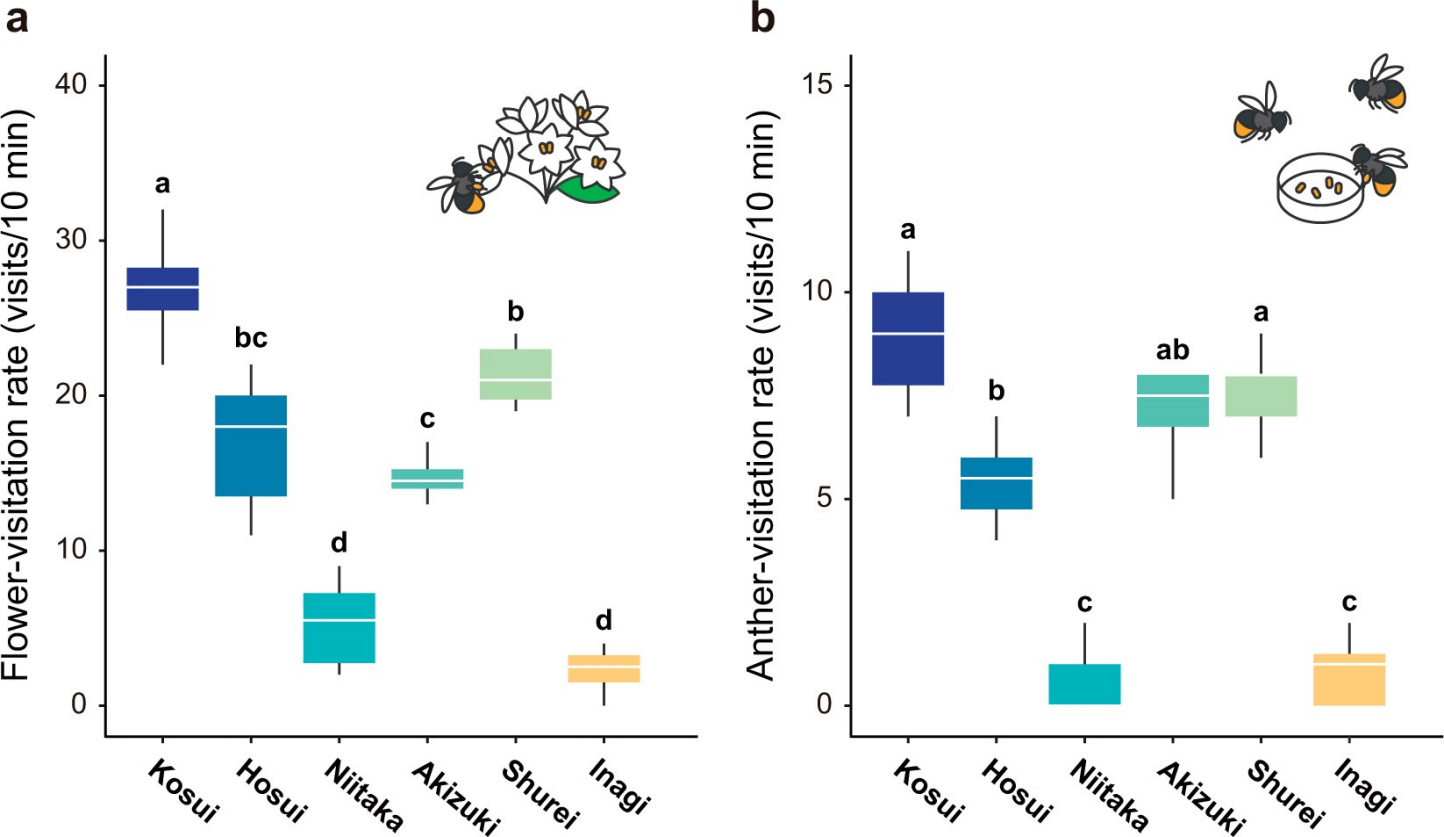
Flower-visiting behavior of bumblebees to the six cultivars of *Pyrus pyrifolia*. The visitation rate of flower-naïve bumblebees, *Bombus ignitus* (**a**) to flowers and (**b**) to pollen-bearing anthers. (**a, b**) Kruskal–Wallis ANOVA (*p* < 0.001) followed by a post-hoc test; different letters denote significant differences in Steel–Dwass multiple comparisons (*p* < 0.05, n = 8). White line, median; whiskers, max & min.

All statistical analyses were performed with R version 4.1.2 [24]. The fruit sets of ‘Akizuki’ and ‘Niitaka’were compared by Mann–Whitney *U* test. The flower-visitation rate was analyzed by generalized linear model (GLM) to estimate the effect of solar illuminance on the flower-visitation rate. The analysis was conducted with the visitation rate as a dependent variable incorporating a Poisson distribution and log-link function. The cultivars were included as independent variables. Kruskal–Wallis ANOVA followed by Steel–Dwass post-hoc test were used to test whether visitation rates (dependent variable) were significantly associated with cultivars (independent variable). Poisson regression models were fitted using the *glm* function to analyze the association of the anther-visitation rates with the protein contents and with P/L, whose fits were assessed by Akaike information criterion (AIC) score. Nagelkerke’s R^2^ values were calculated with the *performance* package [31]. Significant differences in the pollen germination rate and color variables among cultivars were tested by Tukey–Kramer test using the *multcomp* package [32], assuming a normal distribution. *p* < 0.05 was considered statistically significant. The flower-visitation rate was analyzed by generalized linear model (GLM) to estimate the effect of color variables on the flower-visitation rate, with the rate as a dependent variable incorporating a Poisson distribution and log-link function and the variables as independent variables. Relationships between P/L and each color variable were analyzed by linear model. The boxplot in Fig 2 was rendered using the *ggplot2* package [33].

## Results

### Field investigation on the fruit set of bee-pollinated pears

The fruit sets were 93.3% for the male-fertile cultivar ‘Akizuki’ and 44.2% for the male-sterile cultivar ‘Niitaka’. The inflorescences produced one or more fruits were also significantly greater in ‘Akizuki’ (47.8 ± 27.5%) than in ‘Niitaka’ (14.7 ± 22.2%) (Mean ± SD; *p* < 0.001 in Mann–Whitney *U* test).

### Flower-visiting behavior of *B*. *ignitus* to pear cultivars

The illuminance during the assay had no significant effect on bee visitation rate (*F*_1,41_ = 1.102, *p* = 0.299), whereas the cultivar did (*F*_5,41_ = 39.09, *p* < 0.001); therefore, visitation rates were simply compared among cultivars without considering illuminance. Flower-naïve *B*. *ignitus* visited ‘Kosui’ most frequently (26.9 ± 3.1 visits/10 min), followed by ‘Shurei’ (21.3 ± 2.0), ‘Hosui’ (16.9 ± 4.1), ‘Akizuki’ (14.8 ± 1.3), ‘Niitaka’ (5.3 ± 2.7), and ‘Inagi’ (2.3 ± 1.6) (Fig 2A). Very low visitation rates were observed for ‘Niitaka’ and ‘Inagi’ (*p* < 0.05 against other cultivars in Steel–Dwass multiple comparisons), even though bees were flying out of the hive. Bees were noted to collect pollen and nectar from the flowers of ‘Kosui’, ‘Shurei’, ‘Hosui’, and ‘Akizuki’, producing pollen load, but rarely or never collected pollen from ‘Niitaka’ and ‘Inagi,’ and only consumed nectar.

We then carried out another behavioral assay using dehisced anthers (including pollen) (Fig 2B). The same inter-cultivar difference in visitation frequency was seen: bees visited the anthers of ‘Kosui’, ‘Hosui’, ‘Shurei’, and ‘Akizuki’ with a frequency of 5–9 visits/10 min, whereas the visitation rate was less than 1 for ‘Niitaka’ and ‘Inagi’. This assay clearly indicates that bees preferred anthers and pollen of male-fertile cultivars.

### Pollen morphology

**Fig 3:**
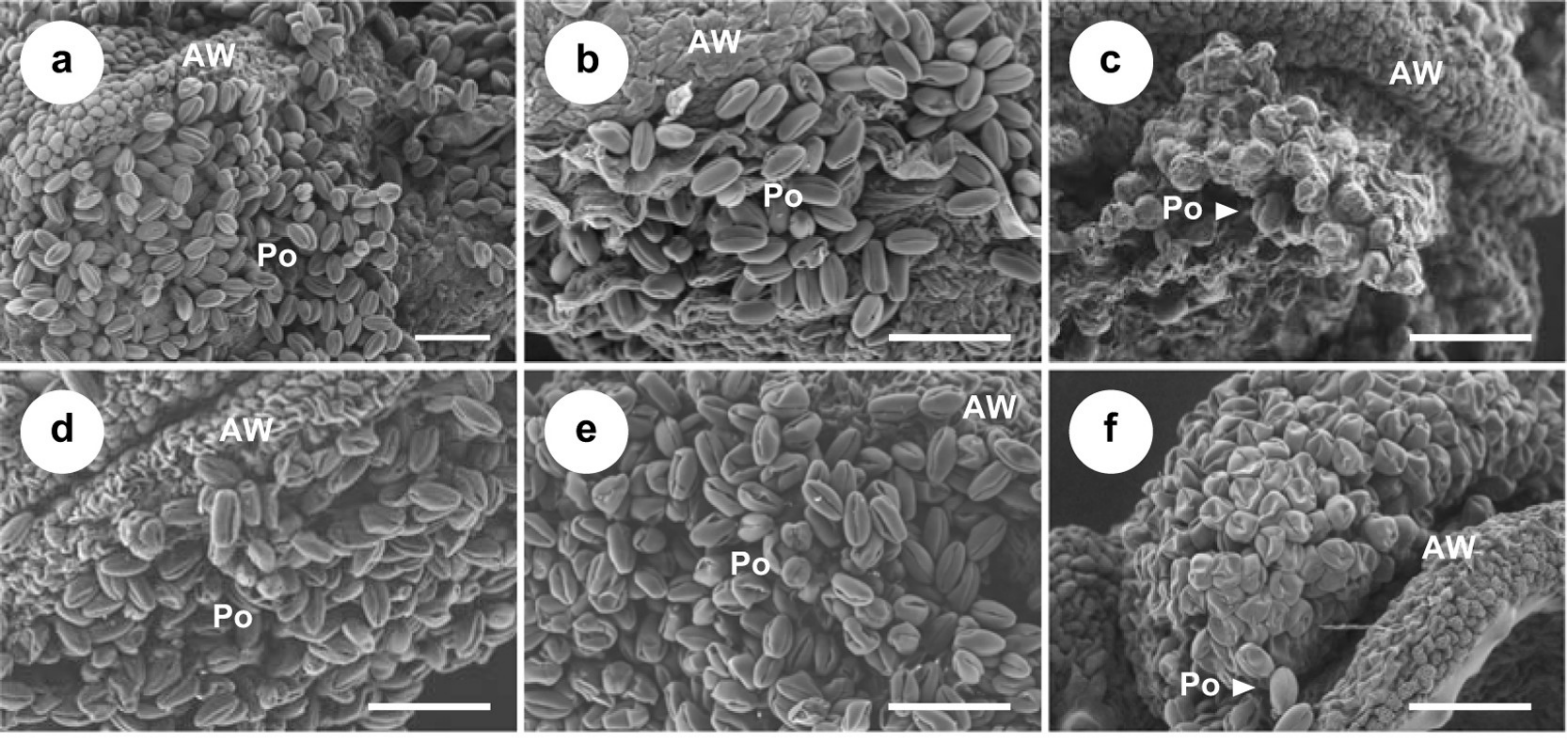
Scanning electron microscopic images of the six cultivars of *Pyrus pyrifolia*. SEM micrographs of anthers (*left*) and magnified pollen grains (*right*) of (**a**) ‘Kosui’, (**b**) ‘Hosui’, (**c**) ‘Niitaka’, (**d**) ‘Akizuki’, (**e**) ‘Shurei’, and (**f**) ‘Inagi’. (**c, f**) Arrowheads indicate morphologically normal pollen. AW, anther wall; Po, pollen. Scale bars: 100 μm.

The pollen-collecting behavior of bees in the behavioral assay inferred some difference in pollen among cultivars. The morphology of the pollen from each cultivar was observed by SEM (Fig 3 and Table 1). Based on the polar axis/equatorial diameter (P/E) ratio of pollen grain [20], the pollen shape of ‘Kosui’, ‘Hosui’, ‘Shurei’, and ‘Akizuki’ were categorized as prolate (P/E = 1.33–2.00) or perprolate (P/E > 2.00). These P/E ratios are similar to those of *P*. *pyrifolia* ‘Xuehuali’, ‘Yali’, ‘Chuwhangbae’, and ‘Imamuraaki’, reported in Nam et al. (2019) [34]. In contrast, almost all pollen obtained from ‘Niitaka’ and ‘Inagi’ was much smaller than the other cultivars, abnormally collapsed, and firmly attached to each other.

**Table 1:** Pollen size and germination rate of six Japanese pear cultivars.

Cultivar	Pol (μm)	Eq (μm)	P/E	Shape type	Germination rate (%)	
Kosui	43.1 ± 2.1	21.5 ± 1.5	1.94 ± 0.12	prolate	66.7 ± 2.6	ab
Hosui	43.5 ± 2.2	22.5 ± 1.3	2.01 ± 0.13	perprolate	56.6 ± 3.1	c
Niitaka	-	-	-	-	12.3 ± 1.1	e
Akizuki	44.9 ± 3.7	22.9 ± 1.3	1.96 ± 0.14	prolate	60.2 ± 4.5	bc
Shurei	43.8 ± 2.1	22.5 ± 2.0	1.96 ± 0.15	prolate	68.8 ± 2.4	a
Inagi	-	-	-	-	20.2 ± 1.9	d

Cultivars other than those from ‘Niitaka’ are male-fertile cultivars. Pol, polar axis; Eq, equatorial diameter. Mean ± SD (n = 10 grains for the size measurement; n = 3 tests with grains > 100 for the germination rate). Different letters denote significant differences in Tukey–Kramer multiple comparisons (*p* < 0.05).

### Pollen viability

The male-fertile cultivars (‘Kosui’, ‘Hosui’, ‘Akizuki’, ‘Shurei’) showed a germination rate of approx. 60%, much higher than the rates of ‘Niitaka’ and ‘Inagi,’ which were 20.2% and 12.3%, respectively (Table 1). The germination rate of ‘Niitaka’ was reported to be 15% [16], consistent with our result. On the other hand, the pollen germination rate of ‘Inagi’ was 76.5% in Takemura et al. (2022) [35], suggesting that the ‘Inagi’ flowers used in that study had undergone abnormal pollen development, possibly caused by (a)biotic stresses. Thereafter, we classified ‘Inagi’ as a male-sterile cultivar as well as ‘Niitaka’. The germination rates positively correlated with anther-visitation rates in the previous section (Nagelkerke’s R^2^ = 0.97 in Poisson regression model); but should not influence bee foraging activity.

### Pollen quality as a reward for *B*. *ignitus*

**Fig 4:**
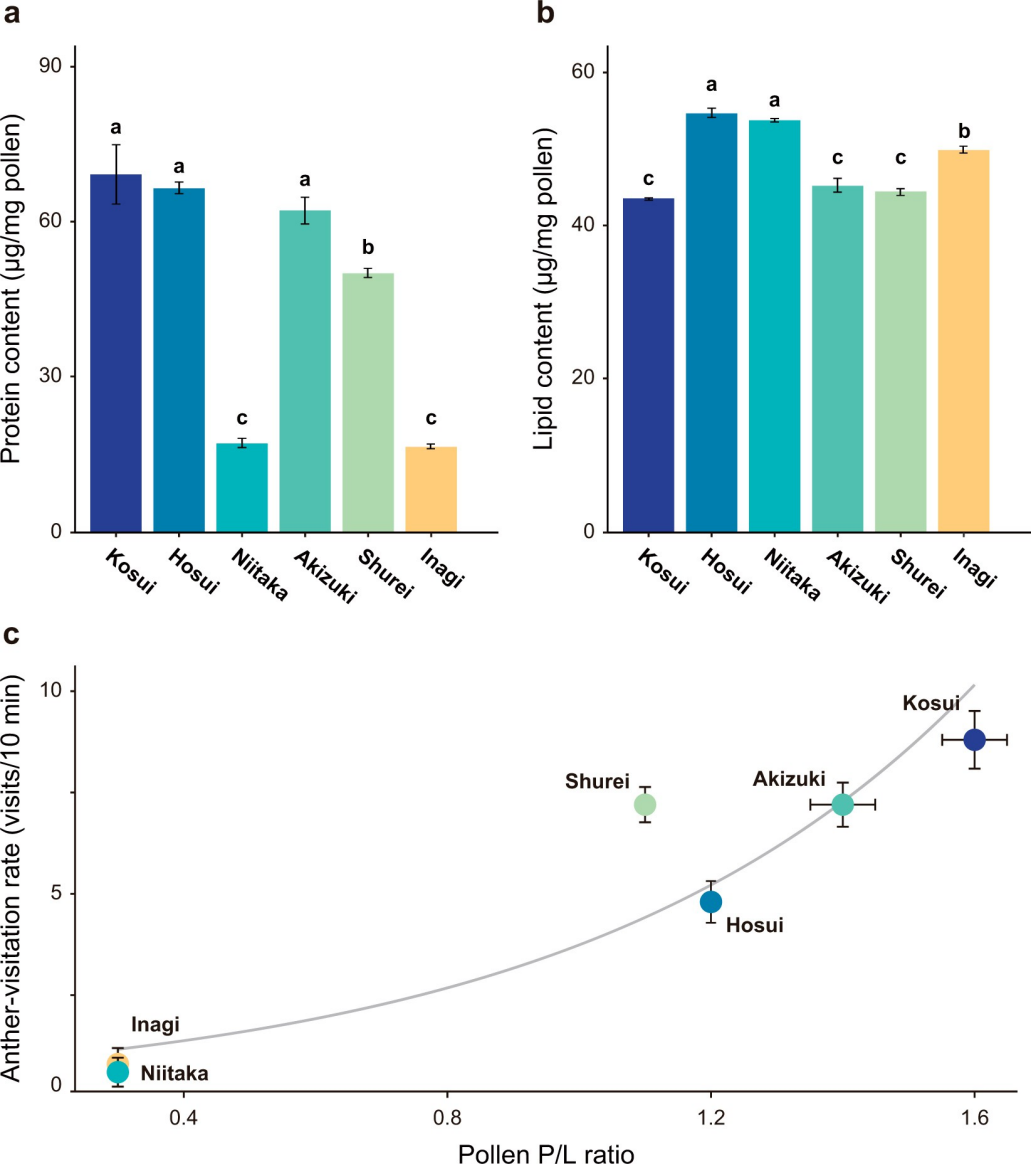
Pollen quality and its relation with the bee anther-visitation rate. Total content of (**a**) proteins and (**b**) lipids in pollen of the six cultivars of *Pyrus pyrifolia*. (**a, b**) Mean ± SD (n = 3). Different letters denote significant differences in Tukey–Kramer multiple comparisons (*p* < 0.05). (**c**) Relationship between P/L ratio and anther-visitation rate of bumblebees (*B*. *ignitus*) towards the six cultivars. Error bars indicate SD of the P/L ratio and visitation rate. Nagelkerke’s R^2^ = 0.96 in Poisson regression model.

The total protein and lipid content in the pollen of each cultivar were quantified (Fig 4), to investigate the link between pollen nutritional value and bee preference. Although pollen of the male-fertile cultivars (‘Kosui’, ‘Hosui’, ‘Akizuki’, ‘Shurei’) contained proteins of 50–70 μg/mg, the protein in the male-sterile pollen (‘Niitaka’, ‘Inagi’) was only less than half of those (< 20 μg/mg). Lipid content ranged from 40 to 55 μg/mg pollen in all cultivars, with little inter-cultivar differences. The resulting P/L of ‘Kosui’, ‘Hosui’, ‘Akizuki’, and ‘Shurei’ was in the range of 1.1–1.6 (Fig 4C; Mean ± SD, 1.3 ± 0.2). In these four male-fertile cultivars, the contents of protein and lipid and the P/L were close to the average of other Rosaceae species (P/L = 1.6 ± 0.3; Vaudo et al., 2020). However, the P/L of both ‘Niitaka’ and ‘Inagi’ were 0.3 ± 0.0, significantly lower compared to the male-fertile cultivars (*p* < 0.001 in Tukey–Kramer multiple comparisons). The anther-visitation rate was positively correlated with protein content in a Poisson regression model (Nagelkerke’s R^2^ = 0.93, AIC = 77.2), but was more highly correlated with the P/L (Nagelkerke’s R^2^ = 0.96, AIC = 71.5). The biased visitation of bees towards male-fertile/sterile cultivars may be mediated by the P/L and possibly by other pollen-derived cues associated with the P/L.

### Colorimetry

**Fig 5:**
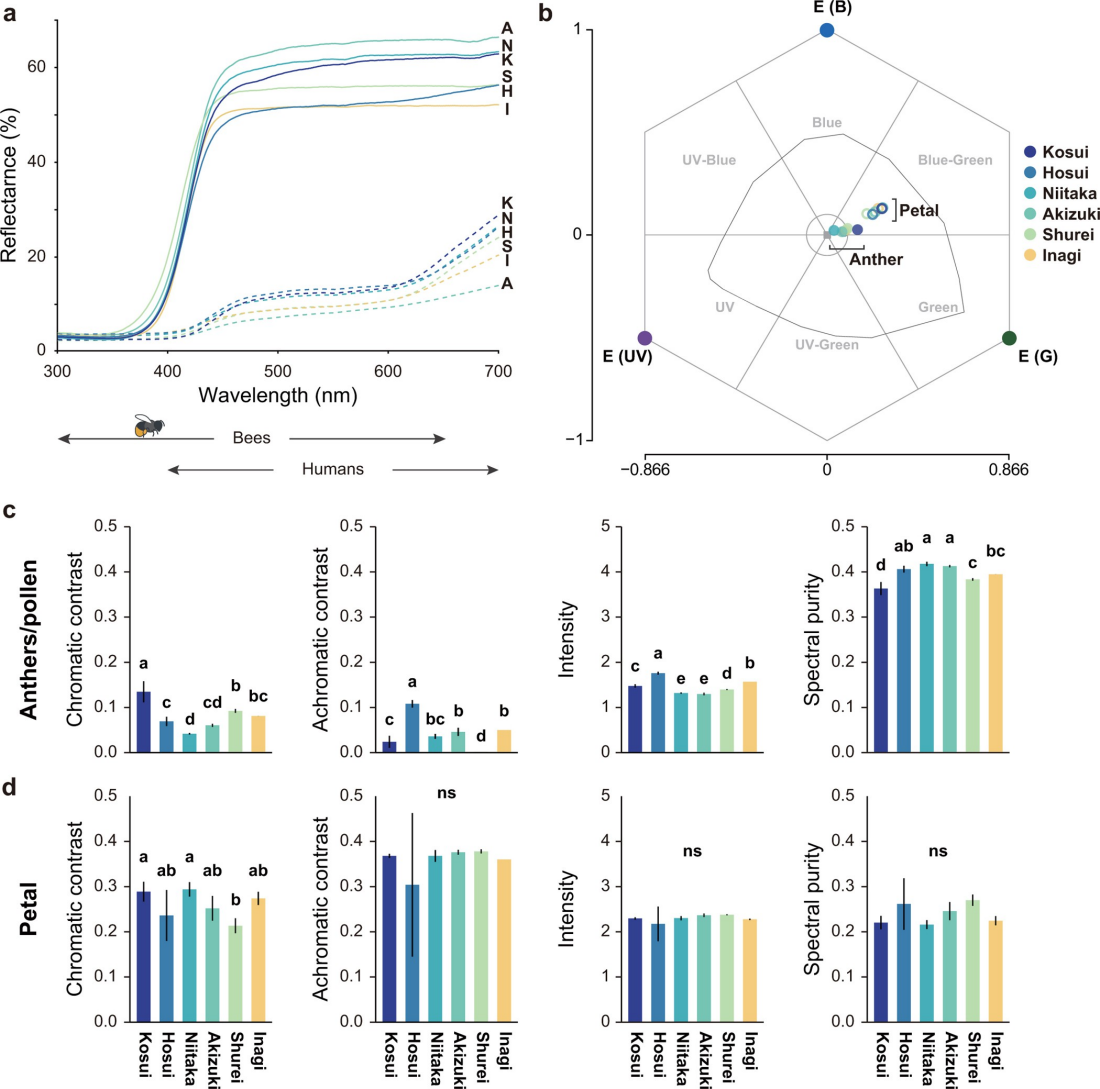
Reflectance of flower organs and their plots in the bee color space. (**a**) Diffuse reflectance spectra of flower organs of the six cultivars: ‘Kosui’ (K), ‘Hosui’ (H), ‘Niitaka’ (N), ‘Akizuki’ (A), ‘Shurei’ (S), and ‘Inagi’ (I). Petals (solid lines) and anthers including pollen (dashed lines). Arrows below the spectra indicate the visible range of bees and human beings. (**b**) Color loci of flower organs plotted in the visual spaces of bees. Petals (filled circles) and anthers including pollen (opened circles). The visual modeling was based on CH model [26]. The curved line represents the spectral locus of theoretical pure stimuli for *Apis mellifera* [29]. The central circle (< 0.1 hex units) encloses the uncolored category that appears achromatic for bees [37]. The box in the center of the hexagon indicates the achromatic center (foliage as a background). The excitation of UV, blue, and green is indicated with respective points in the hexagon space. The spectra and loci represents the average of five biological replications for each cultivar. Color variables of (**c**) petals and (**d**) anthers including pollen in the CH model. Mean ± SD (n = 5 for each cultivar). Different letters denote significant differences in Tukey–Kramer multiple comparisons (*p* < 0.05); ns, not significant.

Since bees possess different visual systems from human beings, they sense the colors differently from human observers. Considering the visible range of bumblebees (300–650 nm), inter-cultivar color differences of anthers and petal were analyzed by their diffuse reflectance spectra (Fig 5A). The reflectance spectra of anthers of the different cultivars were similar, having λ_Max_ over 700 nm with secondary peak at approx. 500 nm. The spectra of the petals of the cultivars were also similar, showing approx. 50–70% reflectance above 450 nm. The reflectance spectra of these petals were almost consistent with the average reflectance spectra of melittophilous flowers reported in Lunau et al. (2011) [36].

Using the reflectance spectra, visual modeling was performed to examine whether bees can discriminate inter-cultivar difference by visual cues. The color loci of the anthers and petals in the bee color hexagon are depicted in Fig 5B. According to the color loci in the bee color hexagon, the anther and petal were classified as Blue or Blue-Green, in close proximity among cultivars. The color loci of the anthers and petals are almost overlapped each other among cultivars, suggesting them to be indistinguishable to bees.

All color variables in the CH models are summarized in Fig 5C and 5D. For anthers including pollen, none of color variables had significant effects on the flower-visitation rate (GLM: chromatic contrast, *p* = 0.832; achromatic contrast, *p* = 0.533; intensity, *p* = 0.488; purity, *p* = 0.683). Significant differences among cultivars were found in all variables (Fig 5C), but they did not correlate with P/L (LM: chromatic contrast, R^2^ = 0.126; achromatic contrast, R^2^ = 0.004; intensity, R^2^ = 0.009; purity, R^2^ = 0.165). For petals, all variables showed significant effects on the flower-visitation rate (GLM: *p* < 0.001). Significant differences among cultivars were detected in chromatic contrast, but not in the other variables (i.e., achromatic contrast, intensity, spectral purity). The chromatic contrast was positively correlated with P/L in LM but rather weak (R^2^ = 0.331).

## Discussion

Our caged-behavioral assay clearly shows the preference of *B*. *ignitus* for the male-fertile cultivars (‘Kosui’, ‘Hosui’, ‘Akizuki’, ‘Shurei’) of *P*. *pyrifolia* compared with the male-sterile cultivars (‘Niitaka’, ‘Inagi’) (Fig 2A). The bees might behave differently in the caged-experiments compared with the natural orchard due to the limited space in the flight cage and differences in the number of accessible plant species. Nevertheless, the low fruit rate of ‘Niitaka’ in the field investigation can be explained with reference to the bias in visitation seen in this assay. Previous studies have reported that *B*. *terrestris* and *A*. *mellifera* also do not prefer ‘Niitaka’ [7,38].

Notably, ‘Inagi’ is an inherently male-fertile cultivar [35], whose flowers are rarely visited by bees when they were abnormally male-sterile. Although the bee visitation rate of normally developed pollen of ‘Inagi’ was not ascertained in this study, these results clearly denote that even male-sterile cultivars are less attractive to bees if their pollen viability has been reduced. Pollen development including microsporogenesis and microgametogenesis is sensitive to (a)biotic stress (i.e., pathogen infection, high or low temperatures, salt stress, water deficit; reviewed in de Storme and Geelen, 2014) [39]. For instance, in ‘Kosui’, the flower bud in the scale-separation stage bearing pollen within the developmental stage from pollen mother cell to tetrad is the most sensitive to cold stress, to which pollen exposed is significantly reduced viability [40]. Since the sensitive period and sensitivity differ among cultivars [40], low temperatures at a certain period can induce the abortion of pollen development to a sensitive cultivar in mixed orchards. When such stresses lead a cultivar to bear abnormally sterile pollen, the cultivar would no longer play a role as a pollinizer, and bees may not visit its flowers. Thereby, the pollination rates of stressed cultivars will be also reduced, lowering the fruit set.

Flowers exhibit a myriad of cues, including floral color, display size, morphology, and odors, all of which can influence and reinforce foraging decisions of bees [41]. For floral colors, the petals of all analyzed cultivars showed chromatic contrast > 0.2 hex units whereby bees can discriminate from leaf background [37]. However, significant differences were detected only between ‘Kosui’–‘Shurei’ and ‘Niitaka’–‘Shurei’. Therefore, differences in petal color are unlikely to be responsible for the difference in flower-visitation rate among cultivars (Fig 5D). For anthers with pollen, the chromatic contrasts were less than 0.1 for all cultivars except ‘Kosui’, suggesting that all the anthers used in this study other than those of ‘Kosui’ appear achromatic to bees. Small inter-cultivar differences were detected in other variables (i.e., achromatic contrast, intensity, spectral purity), but none of them explained the almost exclusive visitation towards the male-fertile pollen (Fig 2C). There also appeared to be little difference in flower morphology and display area among the *P*. *pyrifolia* cultivars tested (Fig 1). Other studies have addressed some inter-cultivar differences in odor and nectar of *P*. *pyrifolia*. For example, Li et al. (2022) compared the profile of flower odor bouquets among cultivars of *P*. *pyrifolia* (‘Golden’, ‘Brown peel’, ‘Xizilü’) by head space SPME-GC-MS [42]. However, whether the odors of male-fertile/sterile cultivars are different is uncertain. Seo et al. (2019) evaluated the nectar of *P*. *pyrifolia* ‘Niitaka’ and ‘Whangkeumbae’ (both of which are male-sterile) and their effects on the foraging activity of *A*. *mellifera*, and concluded that honeybees forage twice as much on ‘Whangkeumbae’ than on ‘Niitaka’ primarily due to the difference in the amino acid composition (Phe, Gly, Ala) and sugar content (sucrose) in the nectar [38]. Such inter-cultivar difference in nectar might be greater between male-fertile and -sterile cultivars.

The preference of bees for male-fertile/sterile flowers was also observed in the anther-visitation rates (Fig 2B)–the bees preferred to visit the anthers of ‘Kosui’, ‘Hosui’, ‘Akizuki’, and ‘Shurei’ over ‘Niitaka’ and ‘Inagi’ even when all the other organs such as the petals had been removed. The anther-visitation rate indicates that the pollen-derived cue plays a role in foraging behavior of bees in the male-fertile/sterile cultivars of *P*. *pyrifolia*. The pollen of ‘Niitaka’ and ‘Inagi’ with low germination rate (≤ 20%) *in vitro* was found to contain significantly less protein (< 20 μg/mg) than that of the male-fertile cultivars (50–70 μg/mg), whereas the lipid content varied little between cultivars (40–50 μg/mg) (Table 1 and Fig 4). The P/L was positively correlated higher than just protein content with bee visitation rate, suggesting its involvement in bees’ preference (Fig 4C). Bumblebees (*B*. *terrestris*) appear to assess protein content by antennating pollen via tactile chemoreceptors [43,44]. They ingest some pollen while foraging or after returning to the hive, and the nutritional information they glean can influence their post-ingestive choice behavior [12,45]. This suggests that the preference of bees for male-fertile pollen arises as a result of chemotactile (antennation) and gustatory (after intake) cues. This may contribute to the preference of bees for the male-fertile cultivars. However, the flower-naïve bees hardly visited either the flowers of male-sterile cultivars or their anthers, suggesting that bees decide not to visit male-sterile flowers/anthers before reaching them. It is possible that visual and/or olfactory cues also influence the bees’ behavior, although color is unlikely to be a factor, as discussed above. Given that flower-naïve bees were able to pre-assess the pollen quality (Fig 2A), their preference for the distant-sensible cue from pollen is likely to be innate. Pollen might have olfactory cues [10], and bees may be able to pre-assess P/L from a distance. Considering that bees prefer male-fertile flowers over male-sterile flowers in many plant species, a common odor characteristics may lie in male-sterile pollens.

## Conclusions

The pollen of male-sterile cultivars had a significantly lower protein content compared with male-fertile cultivars, but their lipid contents were similar, and the P/L of these cultivars highly correlated with the visitation rate of *B*. *ignitus*. The biased flower visitation of *B*. *ignitus* to male-fertile cultivars over male-sterile cultivars may be driven by P/L. However, a few questions remain: (1) how do the bees assess P/L; (2) does P/L target differ depend on bee species, caste, and life stage; and (3) what other nutrient factors influence feeding preference. Petal and anther color is unlikely to be involved in P/L recognition in male-fertile/sterile system. Additional studies of other factors (e.g., flower volatiles, nectar composition, etc.) are necessary to investigate the mechanism underlying the P/L recognition. Under the ongoing climate change, the effects of environmental stress on pollen development and quality as rewards for bees is especially important. Furthermore, pollen with low germination rates had low P/L. The pollen viability of fruiting cultivars is often regarded less important in the cultivation of self-incompatible crops, but low pollen viability may reduce bee visitation due to its low P/L, diminishing the efficiency of bee pollination in the agricultural ecosystem. In plant breeding, artificial selection is often established to produce fruits with desirable characteristics, but evaluation of pollen quality is also essential if bee pollination is expected.
